# Liver Transcriptome Response to Heat Stress in Beijing You Chickens and Guang Ming Broilers

**DOI:** 10.3390/genes13030416

**Published:** 2022-02-25

**Authors:** Astrid Lissette Barreto Sánchez, Qiao Wang, Mamadou Thiam, Zixuan Wang, Jin Zhang, Qi Zhang, Na Zhang, Qinghe Li, Jie Wen, Guiping Zhao

**Affiliations:** State Key Laboratory of Animal Nutrition, Institute of Animal Sciences, Chinese Academy of Agricultural Sciences, Beijing 100193, China; asliss_07@hotmail.com (A.L.B.S.); wangqiao01@caas.cn (Q.W.); mamadou.sleam@gmail.com (M.T.); zixuan.w@foxmail.com (Z.W.); zhangjin0913@126.com (J.Z.); zhangq0117@126.com (Q.Z.); zhangna4520@163.com (N.Z.); liqinghe@caas.cn (Q.L.); wenjie@caas.cn (J.W.)

**Keywords:** heat stress, Beijing You, Guang Ming, transcriptome, WGCNA

## Abstract

Heat stress is one of the most prevalent issues in poultry production that reduces performance, robustness, and economic gains. Previous studies have demonstrated that native chickens are more tolerant of heat than commercial breeds. However, the underlying mechanisms of the heat tolerance observed in native chicken breeds remain unelucidated. Therefore, we performed a phenotypical, physiological, liver transcriptome comparative analysis and WGCNA in response to heat stress in one native (Beijing You, BY) and one commercial (Guang Ming, GM) chicken breed. The objective of this study was to evaluate the heat tolerance and identify the potential driver and hub genes related to heat stress in these two genetically distinct chicken breeds. In brief, 80 BY and 60 GM, 21 days old chickens were submitted to a heat stress experiment for 5 days (33 °C, 8 h/day). Each breed was divided into experimental groups of control (Ctl) and heat stress (HS). The results showed that BY chickens were less affected by heat stress and displayed reduced DEGs than GM chickens, 365 DEGs and 382 DEGs, respectively. The transcriptome analysis showed that BY chickens exhibited enriched pathways related to metabolism activity, meanwhile GM chickens’ pathways were related to inflammatory reactions. *CPT1A* and *ANGPTL4* for BY chickens, and *HSP90B1* and *HSPA5* for GM chickens were identified as potential candidate genes associated with HS. The WGCNA revealed *TLR7*, *AR*, *BAG3* genes as hub genes, which could play an important role in HS. The results generated in this study provide valuable resources for studying liver transcriptome in response to heat stress in native and commercial chicken lines.

## 1. Introduction

Global warming is one of the most serious issues facing the Earth, increasing global temperature leading to climate change, which influences human health and causes complications in animal production [1]. In poultry, the optimum temperature for handling most birds is between 18 and 20 degrees Celsius, and an increase in this temperature can negatively affect growth rate, productivity, and other biochemical characteristics [2,3,4]. This rise of temperature combined with humidity and other factors during prolonged hot weather results in heat stress [5,6,7]. Stress is a biological response to the animal’s adaptation to events that cause it to lose its normal physiological state [5]. When a bird’s heat production exceeds its capacity to disperse it to the surrounding environment, it modifies its behavior and physiological homeostasis in response to elevated temperatures to maintain appropriate thermoregulation [5,8,9,10,11]. Heat stress’s influence on chicken production has been thoroughly examined, especially in broiler breeders [12] and laying commercial hens [13,14].

In addition, it has been reported that chickens under environmental stressor conditions display increased levels of reactive oxygen species (ROS) [15,16,17] in broiler breeders [18] and commercial hens [19]. This results in the oxidative stress stage, characterized by the production and release of heat shock proteins (HSP). Under HSP production and release, the host develops self-protection strategies against deleterious cellular effects of ROS [20]. HSP70 accumulation could be employed as a biomarker to detect heat stress injury in this circumstance [21]. Increased superoxide dismutase (SOD) and catalase enzymes, on the other hand, result in a less severe heat-stress response in cells that lack HSP expression [22]. HSP70 levels were observed to be significantly higher in broilers and laying hens subjected to heat stress [23,24]. Moreover, Tan and collaborators reported an upregulation of the activity of antioxidant enzymes in the liver and serum caused by increased ambient temperature [25].

Moderate stress can benefit the body’s immunity, while severe stress impairs development, productivity, reproduction, and also affects the immune system of animals, ultimately resulting in immunological suppression, disease, and death [26]. Modern poultry genotypes have been reported to generate more body heat as a result of their increased metabolic activity [4,27]. Additionally, some studies have shown that heat stress can affect the number of circulating cells, increasing the heterophil/lymphocyte (H/L) ratio [13,28,29]. This is mainly due to the decrease in concentration of lymphocytes and monocytes and increase in heterophils in the circulation, as a result of the increased glucocorticoid concentrations, such as corticosterone, which is generated by activation of the hypothalamic-pituitary-adrenal (HPA) axis [23,30,31,32,33,34].

Transcriptome comparison has recently gained popularity as a way of studying heat stress. It has the potential to shed light on the genetic basis of heat stress [35]. Furthermore, the liver plays an important role in maintaining homeostasis, general metabolism, and protein synthesis by regulating fat and glucose metabolism [36]. Thus, additional molecular and cellular mechanistic investigations using liver transcriptome profiling may be beneficial for characterizing gene expression changes and elucidating the complex genomic response to heat stress, thereby providing additional insight into the genetic regulation of heat tolerance in chickens [7].

Heat stress affects the poultry industry, and it has been extensively studied. However, the genetic mechanisms behind the heat tolerance observed in native chicken breeds remain unclear. Particularly, no study has yet compared Beijing You and Guang Ming chickens in response to heat stress. Therefore, this study was conducted to assess the phenotypical, physiological, and transcriptome profiles differences between Beijing You and Guang Ming chickens in response to heat stress exposition during five days at 33°C (8 h/day). We also performed a Weighted Gene Co-Expression Network Analysis (WGCNA) of the liver transcriptome to identify these two genetically different chicken breeds’ heat stress-related drivers and hub genes. This study provides valuable resources for studying the mechanisms of heat tolerance in native and commercial chicken breeds and may also facilitate the selection and breeding of heat-tolerant chicken lines.

## 2. Materials and Methods

### 2.1. Experimental Population and Design

Beijing You (BY) and Guang Ming No. 2 broiler line B (GM, new broiler breed bred in China) female chickens were used in this study, involving a total of 140 (80 BY and 60 GM) 21 days old birds. On day 22, the chickens were housed in two environmentally controlled chamber rooms (ECCR). The birds from each breed were divided into control (Ctl) and heat stress (HS) groups. In brief, 50 BY and 30 GM chickens were randomly chosen and allocated to the HS group in different cages within the same ECCR, while the remaining birds (30 BY and 30 GM) were assigned to the Ctl group in separate cages within another ECCR. From day 22 to 24, all the chickens from the two groups were raised with the same temperature and humidity settings (24 °C and 60% relative humidity). Next, from day 25 to 29, the HS group was exposed to 33 °C (with 60% relative humidity) for eight hours each day [37,38]. However, the chickens from the Ctl group were maintained at 24 °C of temperature and 60% of relative humidity during the whole experiment (day 25 to 29). The chickens received ad libitum access to water and feed during the entire experiment.

### 2.2. Phenotypes and Sample Collection

At 5 days post heat stress (29 days old), 15 chickens from each breed (BY and GM) and each experimental group (Ctl and HS) were randomly selected for the measure of the body weight (BW), H/L ratio, Superoxide Dismutase (SOD), and total antioxidant capacity (T-AOC). Before slaughtering the chickens, the body weight and H/L ratio were measured. A total of 1 mL of blood per bird were obtained and stored into a 1.5 microcentrifuge tube at room temperature for later isolation and harvest of serum. To measure the H/L ratio, 10 µL of fresh blood was drawn from each bird’s basilic vein using a syringe, a needle, and a micropipette of 10 µL to drop the same blood volume. Blood smears were subsequently air-dried and stained with Giemsa staining [39]. A total of one hundred leukocytes, including heterophils, lymphocytes, and monocytes, were counted using a schematic diagram and a Leica DM500 microscope with a 100× magnification [40,41]. The H/L ratio was calculated by dividing the heterophil cells by lymphocyte cells. Seven to eight serum samples were used to measure the SOD and T-AOC concentrations according to the manufacturer’s protocol, using the Enzyme-Linked Immunosorbent Assay (ELISA) kit (Cusabio Biotech Co., Ltd., Wuhan, China).

### 2.3. RNA Isolation

To isolate the RNA from the liver, the chickens were aseptically opened and eviscerated for collection of the liver (29 samples, 15 BY and 14 GM; 8–7 and 8–6 samples from Ctl and HS, respectively for each breed). Liver samples were aseptically collected using sterile scissors and tweezers, stored in a cryovial tube, snap-frozen in liquid nitrogen, and stored at −80 °C for later RNA extraction. Next, the QIAGEN RNeasy Kit was used to isolate total RNA, and genomic DNA was removed by using the TIANGEN DNase KIT. The purity of the RNA was determined using the kaiaoK5500^®^ Spectrophotometer (Kaiao, Beijing, China), while the integrity and concentration of the RNA were determined using the Bioanalyzer 2100 system’s RNA Nano 6000 Assay Kit (Agilent Technologies, Santa Clara, CA, USA).

### 2.4. RNA-Seq Library Preparation and Analysis

A total of 2 μg of RNA was used as input material for the production of the RNA samples. The transcriptome data was aligned in paired-end mode to the chicken reference genome (Ensembl GRCg6a) using the HISAT2 (Version: 2.2.0) (accessed on 8 February 2021) (https://daehwankimlab.github.io/hisat2/, accessed on 8 January 2022) with default settings, (Source: https://cloud.biohpc.swmed.edu/index.php/s/hisat2-220-source/download, accessed on 8 January 2022). Sequencing libraries were prepared according to the manufacturer’s instructions using the NEBNext^®^ UltraTM RNA Library Prep Kit for Illumina^®^ (E7530L, New England Biolabs, Ipswich, MA, USA), and index codes were added to assign sequences to each sample. The mRNA from the whole RNA was purified using poly-T oligo-attached magnetic beads. In NEBNext First Strand Synthesis Reaction Buffer, fragmentation was carried out using divalent cations at high temperatures (5X). The first strand of cDNA was generated using a random hexamer primer and RNase H, while the second was synthesized using buffer, dNTPs, DNA polymerase I, and RNase H. Purification of library fragments using QiaQuick PCR kits, elution with EB buffer, terminal repair, A-tailing, and adaptor addition were all performed. Finally, the target products were identified, PCR was carried out, and the library was completed. Next, the quality control of the sequencing data was performed using FastQC (version 0.11.5) [42].

### 2.5. Differential Expression Genes Analysis

DESeq2 [43] (Version 18.2.0) in R software was used to assess the differentially expressed genes (DEGs). The *p*-values were obtained from the Wald test and then corrected by multiple tests using Benjamini-Hochberg (BH) method [44]. The significance was set at fold change of |log2| ≥ 1 and *p*-value < 0.05.

### 2.6. Gene Ontology (G.O.) and Kyoto Encyclopedia of Genes and Genomes (KEGG) Pathway Analysis

To investigate the function of DEGs between the Ctl and HS groups in BY and GM chickens, the ClusterProfiler package [45] in R software was used to perform GO and KEGG pathway enrichment analyses. Based on the DEGs obtained from the comparative analysis of Ctl and HS groups from each breed, GO and KEGG enrichment analyses were performed with a *p*-value of 0.05 stated as a threshold for significant enrichment.

### 2.7. Weighted Gene Co-Expression Network Analysis

Based on all samples’ normalized gene expression data, a weighted gene co-expression network analysis was conducted using the WGCNA (version 1.41) package [46] in R software, with some minor modifications. All the samples from the transcriptome analysis were used to perform the weighted gene co-expression network analysis. The Fragments Per Kilobase per Million (FPKM) was utilized as a standardized measurement of transcription abundance to construct a gene expression matrix with a total of 27 samples (14 BY and 13 GM chicken). The genes with low expression were removed by the WGCNA default function. Topological overlap matrix (TOM) was constructed using the step-by-step network construction method (soft-threshold equal to 9 and 6 for BY and GM, respectively), with a minimum module size of 30 for the module detection. Next, we generated a cluster dendrogram including the module colors and merged dynamic. The modules’ colors were merged with 0.40 and 0.35 for BY and GM, respectively. The cluster dendrogram of co-expression network modules was generated using hierarchical clustering of genes based on the 1−TOM matrix. To assess associations of co-expressed gene clusters with treatment, the HS and Ctl groups were assigned nominal values of 1 and 0 respectively, and the association of co-expressed genes with the other traits was also evaluated. High absolute values of gene significance (GS > 0.5) and module membership (MM > 0.5) with a threshold of *p*-value < 0.01 were used to identify the driver genes [47]. Gene co-expression networks were determined using Cytoscape version 3.6.0 [48] with the edges and nodes provided by the WGCNA “exportNetworkToCytoscape” function. Next, the genes with the high weight based on the intramodular connectivity were identified as hub genes [49,50].

### 2.8. Statistical Analysis

The data were analyzed using GraphPad Prism version 8 (GraphPad Software, San Diego, CA, USA) and R version 4.0.5. The phenotypical and physiological data were analyzed in GraphPad Prism version 8 (GraphPad Software, San Diego, CA, USA). Two-way ANOVA with Sidak’s multiple comparisons was used to analyze differences in H/L ratio, body weight, SOD, and T-AOC between breeds and treatment. Comparison of DEGs shared between the two breeds was performed using jvenn, an open source online tool for comparing lists using Venn Diagrams (http://bioinfo.genotoul.fr/jvennc, accessed on 8 January 2022) [51]. The results are expressed as the mean and standard error of the mean (SEM). All significance was declared for *p* < 0.05.

## 3. Results

### 3.1. Effects of Heat Stress on the Phenotypical and Physiological Parameters between Beijing You and Guang Ming Chickens

**Figure 1:**
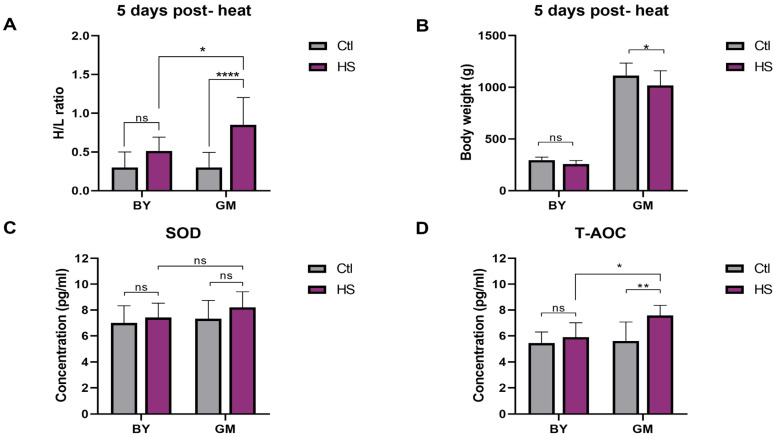
Phenotypical and physiological differences between BY and GM chickens in response to heat stress. (**A**) Heterophils/Lymphocytes (H/L) ratio differences between BY and GM (Ctl, *n* = 13 and HS, *n* = 10). (**B**) Body weight (BW) differences between BY and GM (Ctl, *n* = 13 and HS, *n* = 12). (**C**) Concentration differences of Superoxide Dismutase (SOD) between BY and GM (Ctl, *n* = 7; and HS, *n* = 8). (**D**) Concentration differences of Total antioxidant capacity (T-AOC) between BY and GM (Ctl, *n* = 7; and HS, *n* = 8). All the parameters were measured 5 days post-heat stress. Data analysis was performed using two-way ANOVA, with Sidak’s multiple comparison (α = 0.05). ns (no significant); * (*p* < 0.05); ** (*p* < 0.01); **** (*p* < 0.0001).

To assess the heat stress tolerance between BY and GM, we measured the H/L ratio, body weight (BW), SOD, and T-AOC five days post-heat stress (8 h at 33 °C/day). Chickens from the two breeds were allocated into two environmentally controlled chamber rooms, normal conditions (Ctl group), and stress conditions (HS group). It was noteworthy that the chickens (BY and GM) from the HS group were affected and exhibited heat-related symptoms such as inappetence and respiratory problems. However, BY chickens from the HS group showed less mental depression and heat-related symptoms than GM chickens [52,53]. The results showed that the heat stress increased the parameters studied, except the BW that decreased in the HS group, compared to the Ctl group. The comparison between breeds revealed that GM chickens were more susceptible than BY chickens under heat stress. Moreover, we observed that GM chickens from HS group displayed significantly increased H/L ratio and T-AOC than BY chickens from the HS group (*p* < 0.05; Figure 1A,D, respectively). Remarkably, we observed no significant differences between BY chickens from the Ctl and HS groups in terms of H/L ratio, BW, SOD, and T-AOC (Figure 1A–D, respectively). In contrast, GM chickens from HS group showed significantly increased H/L ratio (*p* < 0.0001) and T-AOC (*p* < 0.01), but significantly decreased BW (*p* < 0.05) than Ctl group (Figure 1A,B,D, respectively).

### 3.2. Liver Transcriptome Sequencing Profiling

In this study, twenty-nine liver samples (15 BY and 14 GM) were used to create transcriptome profiles. The BY chickens included 8 and 7 samples for Ctl and HS groups, respectively. At the same time, for the GM chickens 8 and 6 samples for Ctl and HS groups were used, respectively. The RNA-seq libraries constructed generated more than 42 million raw reads per sample. After filtering and quality control by removing the adaptors and the low-quality reads, more than 40 million clean reads were obtained with an average clean reads rate of 93.09% (Supplement Table S1). Next, the clean reads were aligned to the chicken reference genome (GRCg6a). In total, 23,405 genes were detected among all liver samples. The BY and GM chickens displayed 19,626 (83.85%) and 19,654 (83.97%) expressed genes, respectively.

### 3.3. Identification of Differential Expressed Genes (DEGs) from Beijing You and Guang Ming Chickens between Control and Heat Stress Groups

**Figure 2:**
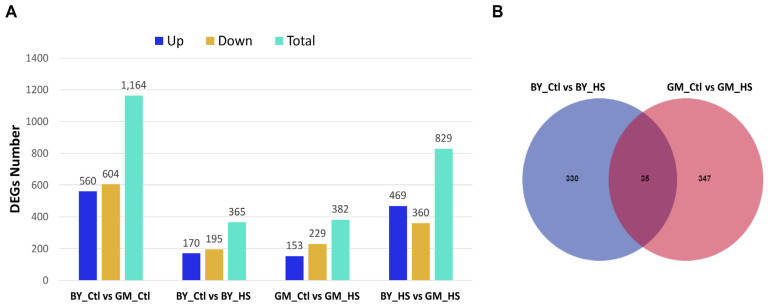
Identification of Differential Expressed Genes (DEGs) in BY and GM chickens. (**A**) Summary of total DEGs between BY and GM in response to heat stress (*p* < 0.05 was used to determine significant DEGs). (**B**) Venn diagram showing the number of unique and shared DEGs between BY and GM in response to heat stress. BY_Ctl: Beijing You control group, GM_Ctl: Guang Ming control group, BY_HS: Beijing You heat stress group, GM_HS: Guang Ming heat stress group.

To determine the DEGs in BY and GM chickens in response to heat stress, transcriptome analysis of liver tissues was performed. In addition, multiple comparisons of DEGs between the two chicken breeds and treatment were performed and visualized using a bar plot (Figure 2A). From the DEGs obtained, we made the following comparison: BY_Ctl vs. GM_Ctl, BY_Ctl vs. BY_HS, GM_Ctl vs. GM_HS, and BY_HS vs. GM_HS (Figure 2A). The results showed more DEGs in the comparison of the two breeds under the same condition (BY_Ctl vs. GM_Ctl and BY_HS vs. GM_HS) than in the comparison of each breed under different conditions (BY_Ctl vs. BY_HS, GM_Ctl vs. GM_HS). In addition, we found that GM chickens (GM_Ctl vs. GM_HS, 382 genes) displayed an increased number of total DEGs, compared to BY chickens (BY_Ctl vs. BY_HS, 365 genes) in response to heat stress (Figure 2A). Moreover, the number of unique and shared DEGs between BY and GM in response to heat stress was also assessed using the Venn diagram (Figure 2B). We found more unique DEGs in GM chickens (347) than in BY chickens (330), with a total of 35 DEGs identified as shared between the two breeds in response to heat stress (Figure 2B). Among the 35 shared DEGs, we can find some genes, such as early growth response 1 (*EGR1*), ethanolamine-phosphate phospho-lyase (*ETNPPL*), NF-kappa-B inhibitor delta-like (*ENSGALG00000028496*), leucocyte cell-derived chemotaxin 2 (*LECT2*), pyruvate dehydrogenase kinase 4 (*PDK4*), solute carrier family 24 member 1 (*SLC24A1*), transcription elongation factor A3 (*TCEA3*). A list of 10 DEGs from each breed is shown in Table 1. The detailed genes information is shown in Supplementary Tables S2 and S3 for BY and GM chickens.

### 3.4. Gene Ontology (GO) Enrichment and Kyoto Encyclopedia of Genes and Genomes (KEGG) Pathways Analysis of Beijing You and Guang Ming Chickens

**Figure 3:**
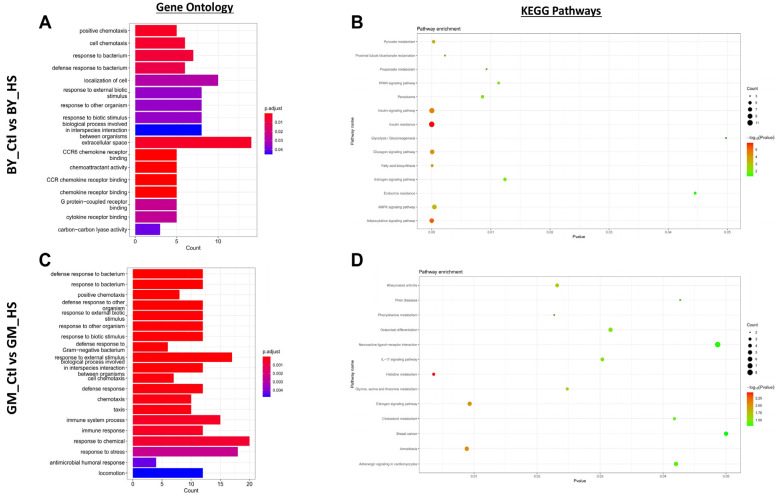
Identification of Gene Ontology (GO) terms and Kyoto Encyclopedia of Genes and Genomes (KEGG) pathways enrichment of Beijing You and Guang Ming chickens in response to heat stress. (**A**) The enriched GO terms are based on the DEGs identified in BY_Ctl vs. BY_HS. (**B**) KEGG pathways enriched based on the DEGs identified in BY_Ctl vs. BY_HS. (**C**) The enriched GO terms are based on the DEGs identified in GM_Ctl vs. GM_HS. (**D**) KEGG pathways enriched based on the DEGs identified in GM_Ctl vs. GM_HS.

Based on the DEGs found in BY (BY_Ctl vs. BY_HS, 365 genes) and GM (GM_Ctl vs. GM_HS, 382 genes) chickens in response to heat stress, GO enrichment, and KEGG pathways were analyzed to assess the effects of heat stress in the two breeds (Figure 3). The results showed that BY and GM chickens displayed approximatively similar numbers and categories, including three domains: biological process, cellular component, and molecular function (Figure 3A,C). The BY chickens exhibited in total 17 GO terms enriched and distributed as follows: (i) under biological process, 9 GO terms were enriched including, positive chemotaxis, cell chemotaxis, response to bacterium, defense response to bacterium, localization of cell, response to external biotic stimulus, response to other organism, response to biotic stimulus, biological process involved in interspecies interaction between organism; (ii) under cellular component, 1 GO term was found as enriched including, extracellular space; and (iii) under molecular function, 7 GO terms were enriched including, CCR6 chemokine receptor binding, chemoattractant activity, CCR chemokine receptor binding, chemokine receptor binding, G protein-coupled receptor binding, cytokine receptor binding, and carbon–carbon lyase activity (Figure 3A). However, GM chickens displayed in total 38 GO enriched terms distributed among (i) biological process, with 26 GO terms such as defense response to other organism, response to biotic stimulus, response to external stimulus, cell chemotaxis, defense response, immune system process, response to chemical, response to stress, humoral immune response, among others were obtained; (ii) cellular components domain, 4 GO terms were enriched including, extracellular region, integral component of plasma membrane, intrinsic component of plasma membrane, extracellular space; and (iii) molecular function with 8 GO terms found as enriched including GO such as cytokine receptor binding, organic acid binding, secondary active transmembrane transporter activity, among others were obtained (Figure 3C).

The KEGG pathways analysis of BY chickens (BY_Ctl vs. BY_HS) showed 14 KEGG pathways significantly (*p* < 0.05) enriched. Pathways related to metabolism activity such as, pyruvate metabolism, proximal tubule bicarbonate reclamation, propanoate metabolism, PPAR signaling pathway, peroxisome, insulin signaling pathway, insulin resistance, glycolysis/gluconeogenesis, glucagon signaling pathway, fatty acid biosynthesis, estrogen signaling pathway, endocrine resistance, AMPK signaling pathway, and adipocytokine signaling pathway were found as significantly enriched in BY_Ctl vs. BY_HS (Figure 3B and Supplementary Table S5). Whereas, for GM chickens (GM_Ctl vs. GM_HS), we found 13 significantly (*p* < 0.05) enriched pathways. The pathways were related to inflammatory reaction, including rheumatoid arthritis, prion diseases, phenylalanine metabolism, osteoclast differentiation, neuroactive ligand-receptor interaction, IL-17 signaling pathway, histidine metabolism, glycine, serine and threonine metabolism, estrogen signaling pathway, cholesterol metabolism, breast cancer, amoebiasis, and adrenergic signaling in cardiomyocytes (Figure 3D and Supplementary Table S5). Remarkably, the analysis of the KEGG pathways showed that the estrogen signaling pathway was the only one shared between the two breeds.

### 3.5. Weighted Gene Co-Expression Network Analysis (WGCNA) of Beijing You and Guang Ming Chickens

**Figure 4:**
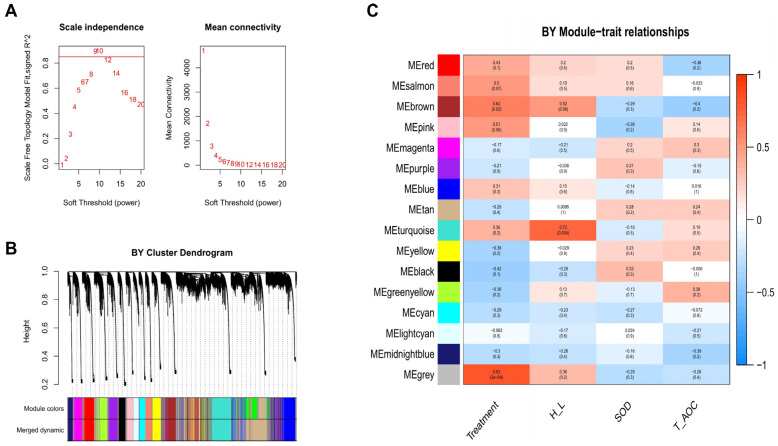
Weighted Gene-Co-Expression Network Analysis (WGCNA) results of Beijing You chickens (liver transcriptomic) show the modules significantly correlated with treatment (heat stress) and blood indicators (H/L, SOD, and T-AOC). (**A**) Scale-free topology model and Mean connectivity. (**B**) Cluster dendrogram of Beijing You chickens reveals the module’s colors and the merged dynamic. (**C**) Heat map of module-trait relationships, each cell has two values. The upper is the absolute value of the correlation coefficient, and down is the *p*-value. Red and blue colors represent positive and negative correlations, respectively.

**Figure 5:**
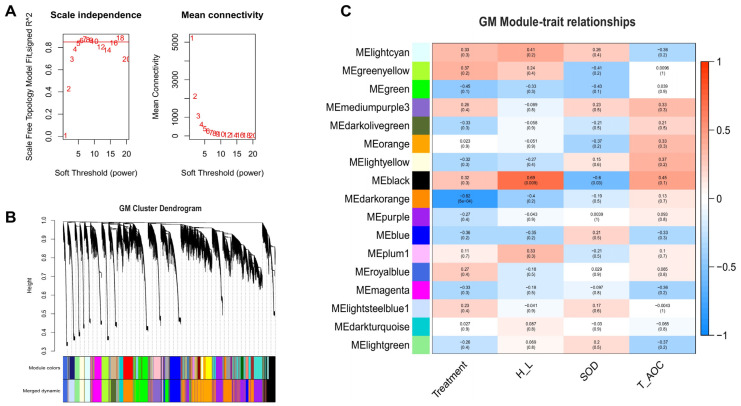
Weighted Gene-Co-Expression Network Analysis (WGCNA) results of Guang Ming chickens (liver transcriptomic) show the modules significantly correlated with treatment (heat stress) and blood indicators (H/L, SOD, and T-AOC). (**A**) Scale-free topology model and Mean connectivity. (**B**) Cluster dendrogram of Guang Ming chickens reveals the module’s colors and the merged dynamic. (**C**) Heat map of module-trait relationships, each cell has two values. The upper is the absolute value of the correlation coefficient, and down is the *p*-value. Red and blue colors represent positive and negative correlations, respectively.

To explore the underlying mechanisms of heat tolerance between BY and GM chickens in response to heat stress, a Weighted Gene-Co-Expression Network Analysis (WGCNA) was performed. This approach can help provide new information related to heat tolerance by identifying the potential driver, candidates, and hub genes associated with heat stress and important blood indicators such as the H/L ratio, SOD, and T-AOC. In this study, the whole liver transcriptome data was used to construct the expression matrix, and the data were analyzed separately for each breed (BY and GM). A total of 19,520 and 19,504 genes were obtained to build the weighted gene co-expression network after removing the offending genes in BY and GM, respectively. First, we determined the best soft threshold 9 and 6 for BY and GM from the scale-free topological model and mean connectivity (Figure 4A and Figure 5A, respectively). Then, using hierarchical clustering of genes based on the 1-TOM matrix, the cluster dendrogram of co-expression network modules were generated (Figure 4B and Figure 5B for BY and GM, respectively). As a result, sixteen co-expression gene modules were established for BY, while seventeen were established for GM. The corresponding modules-trait-relationships are presented in Figure 4C and Figure 5C for BY and GM, respectively.

The grey module of BY chickens (640 genes) was significantly and positively correlated with treatment (r = 0.83, *p* = 2E-04, Figure 4C), while the GM chickens presented a significantly negative correlation between the darkorange module (3388 genes) and the treatment (r = −0.82, *p* = 5 × 10^−4^, Figure 5C). For the H/L trait, both breeds displayed significant positive correlation with this trait. The BY chickens exhibited significant positive correlation of the turquoise module (2272 genes) and brown module (1770 genes) with the H/L ratio (r = 0.72, *p* = 0.004 and r = 0.52, *p* = 0.06, respectively; Figure 4C). However, for the GM chickens we observed that the H/L ratio was positive correlated with lightcyan (r = 0.41, *p* = 0.2) and black (r = 0.69, *p* = 0.009) modules, involving 526 and 1037 genes, respectively. For the SOD trait, BY chickens showed positive correlation (r = 0.33, *p* = 0.3 with the black module (1037 genes), while GM chickens displayed a significant negative correlation (1037; r = −0.6, *p* = 0.03) in the black module (1037 genes; Figure 5C). Finally, the last trait (T-AOC), was positively correlated with greenyellow module (1290 genes; r = 0.38, *p* = 0.2) but negatively correlated with brown module (1770 genes; r = −0.4, *p* = 0.2) for BY chickens while, for GM chickens the black module (1037; r = 0.45, *p* = 0.1) was positively correlated with T-AOC (Figure 5C).

Furthermore, we identified the top five driver genes from each module-trait relationship for BY and GM chickens by filtering the significant genes (*p* < 0.01). After filtering the treatment trait’s genes, we found a total of 62 and 755 significant genes for BY (grey module) and GM (darkorange module) chickens, respectively. In the case of the H/L trait for the BY chickens, the results revealed 407 and 99 significant genes in the turquoise and brown modules, respectively. However, for the H/L ratio trait measured in GM chickens, 193 significant genes were detected in the black module. Concerning the SOD, 4 and 90 significant genes were identified in BY and GM chickens, respectively. Finally, the T-AOC trait evaluated in BY and GM chickens, 9 and 20 significant genes were detected as significant in the greenyellow and black modules, respectively. Next, the top 5 driver genes were selected according to the absolute value of gene significance (GS) and module membership (MM). As a result, the top 5 drivers are presented in Table 2.

### 3.6. Screening of Hub Genes Related to Heat Stress in Beijing You and Guang Ming Chickens

**Figure 6:**
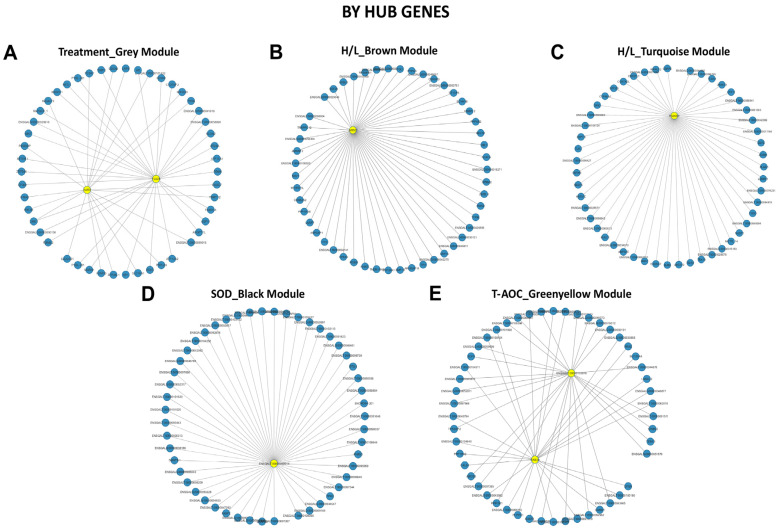
Co-expression network of hub genes in BY chickens: (**A**) Filtered co-expression network treatment grey module. (**B**) Filtered co-expression network H/L brown module. (**C**) Filtered co-expression network H/L turquoise module. (**D**) Filtered co-expression network SOD black module. (**E**) Filtered co-expression network T-AOC greenyellow module.

**Figure 7:**
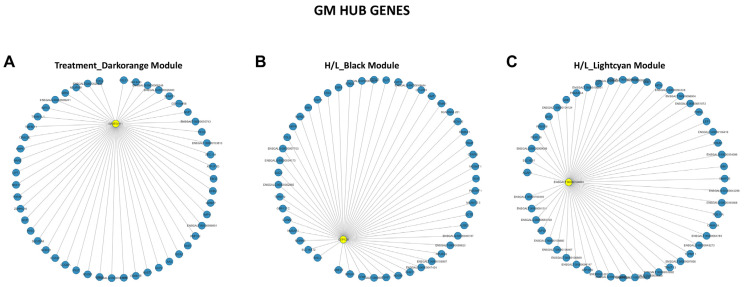
Co-expression network of hub genes in GM chickens: (**A**) Filtered co-expression network treatment darkorange module. (**B**) Filtered co-expression network H/L black module. (**C**) Filtered co-expression network H/L lightcyan module.

The hub gene class is a set of highly connected genes within a module significantly involved in biological functions [54]. First, we filtered the key module by selecting the modules strongly correlated with traits to identify the candidate’s hub genes. The co-expression network with detected hub genes of the significant modules identified for BY and GM chickens is shown in Figure 6 and Figure 7, respectively. The grey (Treatment), brown (H/L ratio), turquoise (H/L ratio), black (SOD), and greenyellow (T-AOC) modules were identified as highly correlated with the studied traits in BY chickens (Figure 6), while the darkorange, black, and lightcyan modules were detected as strongly correlated with Treatment and H/L ratio, respectively in GM chickens (Figure 7). Interestingly, some hub genes were identified as the driver and are presented in Table 2. The treatment trait was significantly and positively (r = 0.83 and *p* = 2 × 10^−4^) correlated with the grey module for BY chickens (Figure 4C), while the darkorange module was identified as significantly and negatively (r = −0.82 and *p* = 5 × 10^−4^) correlated with the treatment in GM chickens (Figure 5C). In the grey module (Figure 6A), we found 2 hub genes, histidyl-tRNA synthetase (*HARS*) and coiled-coil domain containing 130 (*YJU2B*). However, we identified the G-patch domain containing 11 (*GPATCH11*) for the treatment in GM chickens in the darkorange module (Figure 7A). For the H/L ratio, we identified the brown (r = 0.52 and *p* = 0.06, Figure 4C) and turquoise (r = 0.72 and *p* = 0.004, Figure 4C) modules in BY chickens (Figure 6), while the black (r = 0.69 and *p* = 0.009, Figure 5C) and lightcyan (r = 0.41 and *p* = 0.2, Figure 5C) modules were identified as significantly and positively correlated with H/L ratio in GM chickens (Figure 7). From the brown module (Figure 6B), we identified nardilysin convertase (*NRDC*), while for the turquoise module fructosamine 3 kinase-related protein (*FN3KRP*) was identified as hub gene (Figure 6C) in BY chickens. In the black and lightcyan modules, we identified centrosomal protein 128 (*CEP128*) and the transcript *ENSGALT00000106893* as hub genes, respectively (Figure 7B,C, respectively) in GM chickens. Concerning the SOD, we found a module (black) correlated (r = 0.33 and *p* = 0.3, Figure 4C) with this trait only in BY chickens. The *ENSGALT00000060014* transcript in the black module was identified as the hub gene for SOD (Figure 6D). Like the SOD, the T-AOC showed only one module correlated (r = 0.38 and *p* = 0.2) with it in BY chickens, the greenyellow module (Figure 4C). The transcript *ENSGALT00000060014* and member RAS oncogene family (*RAB3B*) were identified as hub genes in Beijing You chicken (Figure 6E).

## 4. Discussion

Climate change has been observed in recent decades due to global warming. High temperatures are one of the major environmental variables causing important economic losses in the agricultural sector and poultry production. Heat stress has been extensively studied as a source of concern for animal growth and development, particularly reproductive efficiency [55,56]. Therefore, a heat stress index must be measured to elucidate the underlying biological mechanisms of heat stress on production performance in poultry. Heat stress-induced alterations in homeostasis can be measured using physiological indices of heat tolerance [57]. Heat stress can also impair the immune system’s effective response to infections, resulting in increased disease severity or death [5]. Previous research has been conducted to investigate the effect of heat stress on the rate of development and production in birds [5,13,30,58,59]. In the present study, we performed a comparative phenotypic, physiological, liver transcriptome analysis, and WGCNA in response to heat stress between BY and GM chickens to determine which of these two breeds displays heat tolerance advantages. The phenotypical comparison revealed that the heat stress induced significant changes in the physiology of the birds and that BY chickens were less affected. In accordance with our results, previous studies reported significant changes induced by the exposition to heat stress [13,29,58].

Heterophil cells are a type of white blood cell found in chickens, which act on innate immunity, immunological defense, and immune control. Heterophils, known as neutrophils in mammals, display functions such as pathogens killing and trapping, phagocytosis, oxidative bursts, cytokine generation, and other mechanisms protecting poultry from the invasion of pathogens [60]. The ROS production of phagocytic leukocytes in response to external stimuli (such as microbes and microorganism-related chemicals) is known as oxidation or respiratory burst. Numerous studies have shown that the H/L ratio is an essential feature of cell-mediated immunity that can be used as an accurate physiological indicator or biomarker to predict heat stress response and disease resistance [41,61,62,63]. The H/L ratio in chicken peripheral blood is widely recognized as a valid and accurate physiological biomarker of stress response in chickens [32,60,61,64,65,66,67]. External stressors (such as bacterial infection, high or low temperature, excessive NH3 exposure, and other stress reactions) decrease the number of lymphocytes and monocytes in the blood while inducing an increase in heterophils number, increasing the H/L ratio [29,63,68,69]. This work found an increased H/L ratio in both breeds, with less effect in BY chickens than in GM chickens. Similarly, we observed the same tendency for the T-AOC blood serum level and the body weight. These results suggest that compared to GM chickens, BY chickens display heat tolerance advantages. Accordingly, a previous study comparing resistance and susceptible chickens to heat stress reported an increase in the H/L ratio.

Body weight is one of the major physiological parameters affected by heat stress in animals. It has been shown that broilers are the most affected by heat stress and display a more pronounced alteration of the growth rate performance [27,70,71,72,73]. Accordingly, we observed that GM chickens (compared to GM_Ctl) displayed significantly decreased body weight under heat stress compared to BY chickens. The results obtained in terms of phenotypical parameters can be explained by the fact that native chickens display more homeostasis regulation mechanisms than commercial chickens. Previous research [74] comparing two chicken breeds, a fast-growing commercial (Arbor Acres) and a slow-growing local breed (Beijing You chicken), reported a decrease in body weight in heat-exposed broilers, while local chicken growth was not affected. The same authors also suggested that the Beijing You breed is more resistant to high-temperature environments.

It is known that exposure to high temperatures induces an increase in ROS, which will stimulate the production of oxidative enzymes, such as SOD and T-AOC [25,75]. However, Willemsen and collaborators found that chronic heat stress in broilers did not alter plasma SOD activity [76]. Therefore, based on blood serum analysis performed in this study, we found no significant effects of heat stress exposition in both breeds in terms of SOD concentration. However, concerning T-AOC blood serum concentration, we found that GM_HS chickens displayed significantly increased T-AOC compared to GM_Ctl chickens. Moreover, we found that for the same indicators, BY chickens showed no significant changes due to heat stress exposition. These results indicate that GM chickens were more affected than BY chickens under heat stress. In contrast, a study carried out with another species of bird reported a decrease in CAT (catalase) and SOD activities in the liver, as well as a decrease in total antioxidant capacity (T-AOC) in Pekin ducks exposed for 1 h to 39 ± 0.5 °C [77]. This difference with our study may be explained by the difference in species, age, temperature, and time of heat stress exposition.

Transcriptome sequencing has been used in several species, such as poultry, cattle, and swine, to identify the genes that play a key role in response to exposition to high environmental temperature [78,79]. In the present study, we performed transcriptome analysis from liver tissue of two different chicken breeds to explore the candidate genes that could play a major role in heat tolerance. The liver is widely recognized as an important organ in sustaining homeostasis when the animal is subjected to high temperatures. In the current work, 365 DEGs, including 195 downregulated and 170 upregulated, were identified in BY liver transcriptome analysis in response to heat stress. Based on DEGs and KEGG pathways analysis, 25 genes were identified (Supplementary Table S5). Among these genes, *PCK1*, *G6PC*, *CPT1A*, *PPARGC1A,* and *ANGPTL4* were identified as candidate genes related to heat stress. Phosphoenolpyruvate carboxykinase (*PCK1*) is a key target for gluconeogenesis control. Insulin, glucocorticoids, cAMP, and nutrition can modulate this gene’s expression to adjust glucose production to physiological requirements [80]. Glucose-6-phosphatase (*G6PC*), a critical enzyme in blood glucose homeostasis regulation, catalyzes the terminal step in gluconeogenesis and glycogenolysis [81]. Carnitine palmitoyltransferase IA (*CPT1A*) is a liver enzyme involved in fatty acid oxidation, and it is critical for energy production [82,83,84]. Due to its enzymatic capabilities and location, it is known as *L-CPT1* or *CPT1A* in mammals and chickens. This gene has been reported to be activated during lack of energy in humans and mice muscles [85,86,87]. *PPARGC1A* gene functions as a coactivator for nuclear receptors and other transcription factors that regulate energy metabolism genes. This protein can interact with and modulate the activity of cAMP response element binding protein (CREB) and nuclear respiratory factors (NRFs). It establishes a direct relationship between external physiological stimuli and the regulation of mitochondrial biogenesis, and is a critical factor in determining the type of muscle fiber. Moreover, this protein is possibly implicated in blood pressure regulation, cellular cholesterol homeostasis regulation, and the development of obesity [88]. The angiopoietin-like 4 (*ANGPTL4*) gene has been identified to play a key role in the process of homeostasis and reactive oxygen species (ROS) [89]. A group of researchers [90] found that the *ANGPTL4* gene was recognized as DEG upregulated in the Fayoumi breed, a local chicken considered a heat-resistance breed. This emphasizes the important involvement of *ANGPLT4* in the function of heat-induced metabolic alterations in the liver. All the genes previously mentioned were related to metabolism activity and the following pathways: insulin resistance, adipocytokine signaling, insulin signaling, glucagon signaling, AMPK signaling, glycolysis/gluconeogenesis, and PPAR signaling pathway, which are activated in response to stress and fatty oxidation.

Concerning GM chickens, 382 DEGs, including 229 downregulated and 153 upregulated, were identified in GM liver transcriptome analysis in response to heat stress. Based on these DEGs and KEGG pathways 38 genes were identified (Supplementary Table S5). Among these genes, *IL1R1*, *HSP90B1,* and *HSPA5* were identified as candidate genes associated to heat stress. *IL1R1* is a member of the interleukin-1 receptor family of cytokine receptors (α, β, and antagonist), a critical mediator in the immunological and inflammatory responses caused by various cytokines [91]. *HSP90B1* is an HSP90 protein found in the endoplasmic reticulum [92]. HSP90 proteins are a family of highly conserved molecular chaperones that play critical functions in signal transduction, protein folding, degradation, and morphological evolution. HSP90 proteins generally accompany other newly generated proteins and the stabilization and refolding of denatured proteins after stress. Heat Shock Protein Family A (Hsp70) Member 5 (*HSPA5*), is a protein localized in the endoplasmic reticulum (ER) lumen, where it acts as a normal HSP70 chaperone, assisting in the folding and assembly of proteins within the ER and acting as a master regulator of ER homeostasis. *HSPA5* interacts with the transmembrane stress sensor proteins. *HSPA5*, also known as glucose-regulated protein 78 (*GRP78*), is a key component of the antioxidant defense mechanism that works towards the end of macroautophagy under stress. These genes were related to inflammatory reactions, and enriched in amoebiasis, estrogen signaling, prion diseases, osteoclast differentiation, and IL-17 signaling pathways.

BY and GM chickens shared only one KEGG pathway, the estrogen signaling pathway. Estrogen plays a critical physiological role in female development and reproductive function during times of stress [93,94,95]. Estrogen regulates the physiological processes of lipid metabolism in chicken liver during the egg-laying cycle [96]. Estrogens have been shown to induce transcription of estrogen-dependent genes and to improve the stability of transcripts [97]. Estrogens act via two estrogen receptors, α (Erα) and β (Erβ) [98]. It is well established that estrogens have a dramatic effect on the neuroendocrine and reproductive behavioral responses to stress, and that these capacities are mediated by the estrogen receptor Erα [99]. In addition, Erα is altered when it is involved in modulating the reproductive function under stressful conditions. Two DEGs (*ADCY1* and *FOS*) were found in this pathway for both breeds. *ADCY1* is the founding member of the adenylate cyclase family of enzymes responsible for the synthesis of cAMP [100]. *ADCY1* gene was also enriched in adrenergic signaling in cardiomyocytes pathway, which is critical in regulating cardiac function in response to environmental changes [101]. *FOS*, *FOSB*, *FOSL1*, and *FOSL2* are the four members of the Fos gene family. These genes code for leucine zipper proteins, which can bind to JUN family proteins to create the transcription factor complex AP-1. As a result, the FOS proteins have been linked to cell proliferation, differentiation, and transformation regulators [102].

Weighted Gene Co-Expression Network Analysis (WGCNA) was performed to explore the underlying mechanisms of heat tolerance between BY and GM chickens in response to heat stress. *BMP15*, *AR,* and *TLR7* were three of the hub genes from BY chickens. *BMP15* is a member of the bone morphogenetic proteins (BMPs) family of multifunctional growth factors that are a subfamily of the transforming growth factor β (TGF-β) superfamily [103]. *BMP15* promotes the proliferation and differentiation of granulosa cells, as well as ovarian folliculogenesis, and appears to be required for female reproduction in mammals [104]. An androgen receptor (*AR*) is a hormone-inducible DNA-binding transcription factor that is required for reproduction through the transmission of androgen signals [105]. *TLR7* is a critical component of innate and adaptive immunity involved in the identification of RNA viruses, particularly highly dangerous ones, such as avian influenza viruses [106]. Another study discovered that members of the Toll-like receptor (TLR) family are required for *Salmonella* detection and clearance [107,108]. *Salmonella*-induced TLR activation results in the generation of inflammatory cytokines and antimicrobial chemicals, including IL-1β, IL-18, IFN-γ, TNF-α, and reactive oxygen species, which are essential mediators for bacterial growth control in host tissues [109]. Moreover, in GM chickens we found *CCNA2*, *UBE2T* and *BAG3* as hub genes. *CCNA2* has been reported to be required in all stages of the mammalian embryonic and somatic cell cycles [110], and functions by forming specialized serine/threonine protein kinase holoenzyme complexes with either *CDK1* or *CDK2* [111]. *UBE2T* catalyzes ubiquitin’s covalent binding to protein substrates. Additionally, this gene is involved in the degradation and ubiquitination of *BRCA1* [112]. *BAG3* acts as a co-chaperone for the chaperone proteins HSP70 and HSC70 [113]. It also acts as a nucleotide-exchange factor (NEF), stimulating the release of ADP from the HSP70 and HSC70 proteins and therefore initiating the release of client/substrate proteins. In vitro and in mammalian cells, the *BAG* domains of *BAG1*, *BAG2*, and *BAG3* selectively interact with the Hsc70 ATPase domain [114]. Taken together these results show that the candidate genes are not only considered as potentially associated with heat stress, but also are relevant for female reproduction and immune system of the host. The candidate genes identified in this study may be beneficial for future studies related to heat tolerance in chickens.

## 5. Conclusions

Heat stress is one of the major environmental stressors affecting poultry production in tempered regions and intensive production systems. This study evaluated the effects of heat stress on phenotypical, physiological, and transcriptomic parameters of two genetically distinct breeds, namely Beijing You and Guang Ming chickens. According to the phenotypical and physiological results, Beijing You chickens were less affected by heat stress than Guang Ming chickens, indicating that this native breed displays heat stress resistance advantages compared to the commercial breed. Based on transcriptome analysis, we found that *CPT1A* and *ANGPTL4* in Beijing You, and *HSP90B1* and *HSPA5* in Guang Ming chickens could be potential biomarkers of heat stress in chickens due to their respective functions. Moreover, *ADCY1* and *FOS* genes were identified in the estrogen signaling pathway, which was shared between the two breeds. Finally, driver and hub genes such as, *TLR7*, *AR*, *BAG3* were also identified. Therefore, this study provides a valuable resource of data sets to study the heat tolerance in native and commercial chicken lines. Furthermore, the driver and hub genes identified in this work could be useful for future molecular study and thus contribute to expanding our current knowledges related to heat tolerance in chickens and the development of heat stress resistant chicken lines.

## Figures and Tables

**Table 1:** DEGs of Beijing You and Guang Ming broiler chicken in response to heat stress (selected).

Beijing You				Guang Ming Broiler			
Ensembl Gene ID	Gene Name	Regulated	*p*-Values *	Ensembl Gene ID	Gene Name	Regulated	*p*-Values *
ENSGALG00000000619	*ANGPTL4*	up	0.047959	ENSGALG00000000112	*PLP1*	down	0.013577
ENSGALG00000000949	*HBEGF*	down	1.43 × 10^−8^	ENSGALG00000000158	*MHCDMA*	down	0.000247
ENSGALG00000001094	*ADGRD2*	up	0.02279	ENSGALG00000000314	*NEFL*	down	0.040557
ENSGALG00000001252	*CREB3L3*	down	9.88 × 10^−10^	ENSGALG00000000318	*CSRP1*	down	0.006843
ENSGALG00000001347	*LHX6*	up	0.040949	ENSGALG00000000556	*UTS2*	up	0.006796
ENSGALG00000001662	*ATP10B*	down	0.030923	ENSGALG00000000667	*EDN2*	down	0.04102
ENSGALG00000001723	*-*	up	0.040431	ENSGALG00000000901	*-*	up	0.000927
ENSGALG00000001749	*ACSBG2*	up	0.000153	ENSGALG00000001000	*HSPA5*	up	2.31 × 10^−5^
ENSGALG00000001963	*-*	down	0.019399	ENSGALG00000001115	*MMEL1*	up	0.008385
ENSGALG00000002028	*GPRC5B*	down	4.84 × 10^−6^	ENSGALG00000001136	*-*	up	0.048206

* *p*-values were obtained by BH method.

**Table 2:** Top five driver genes in the significant modules of Beijing You and Guang Ming chickens 5 days post-heat stress.

Trait	Breed	Correlation	Module Color	Gene Names (GS, MM)
Treatment	Beijing You	Positive	Grey	*HARS (0.89, 0.77), TRMT12 (0.86, 0.69), ENSGALG00000004144 (0.85, 0.78), B4GALT4 (0.83, 0.80), MSI1 (0.82, 0.88)*
	Guang Ming	Negative	Darkorange	*POLR2I (−0.95, 0.93), C1orf232 (−0.94, 0.81), GGACT (−0.93, 0.88), MIF4GD (−0.92, 0.73), ECD (−0.92, 0.81)*
H/L	Beijing You	Positive	Turquoise	*OMG (0.88, 0.80), ENSGALG00000030007 (0.88, 0.70), LONRF1 (0.88, 0.69), BRI3BP (0.87, 0.83), MMP13 (0.87, 0.72)*
			Brown	*ENSGALT00000045744 (0.91, 0.64), SEC61A2 (0.83, 0.75), UGT1A1 (0.83, 0.82), RD3L (0.82, 0.76), ENSGALT00000101291 (0.82, 0.76)*
	Guang Ming	Positive	Black	*ENSGALG00000053515 (0.91, 0.79), CILP (0.87, 0.61), ENSGALT00000106076 (0.87, 0.79), ENSGALT00000028967 (0.86, 0.83), ENSGALG00000048900 (0.86, 0.82)*
SOD	Beijing You	Positive	Black	*ENSGALG00000031869 (0.76, 0.50), CNIH3 (0.74, 0.58), ENSGALT00000053113 (0.67, 0.66), RTCA (−0.75, −0.49)*
	Guang Ming	Negative	Black	*NCAPG2 (−0.88, 0.76), ENSGALT00000098242 (−0.87, 0.77), UBE2T (−0.87, 0.57), ESCO2 (−0.85, 0.62), OVALX (−0.85, 0.62)*
T-AOC	Beijing You	Positive	Greenyellow	*ENSGALG00000046741 (0.77, 0.62), DPH2 (0.76, 0.59), MEF2A (0.74, 0.44), ENSGALG00000047728 (0.73, 0.40), ENSGALG00000019352 (0.72, 0.71)*
	Guang Ming	Positive	Black	*RUFY3 (0.83, 0.62), ENSGALG00000051746 (0.81, 0.59), ENSGALG00000037214 (0.80, 0.43), GALNT11 (0.79, 0.061), CEP112 (0.77, 0.53)*

Note: GS, Gene Significance; MM, Module Membership.

## Data Availability

The datasets generated during and/or analyzed during the current study are available from the corresponding author on reasonable request.

## Supplementary Materials

The following supporting information can be downloaded at: https://www.mdpi.com/article/10.3390/genes13030416/s1, Figure S1: Module Membership vs. Gene Significance of the different traits of Beijing You chicken; Figure S2: Module Membership vs. Gene Significance of the different traits of Guang Ming chicken; Table S1: RNA sequencing Summary; Table S2: Differential Gene Expression data for each library—Beijing You chicken; Table S3: Differential Gene Expression data for each library—Guang Ming chicken; Table S4: Differential Gene expression shared between the two breeds; Table S5: Significant GO terms and KEGG pathways of Beijing you and Guang Ming chicken.
