# Supplementary figures and images for: Liver Transcriptome Response to Heat Stress in Beijing You Chickens and Guang Ming Broilers
Source: Genes (Basel). 2022 Feb 25;13(3):416. doi: 10.3390/genes13030416 (PMC8953548; doi:10.3390/genes13030416)

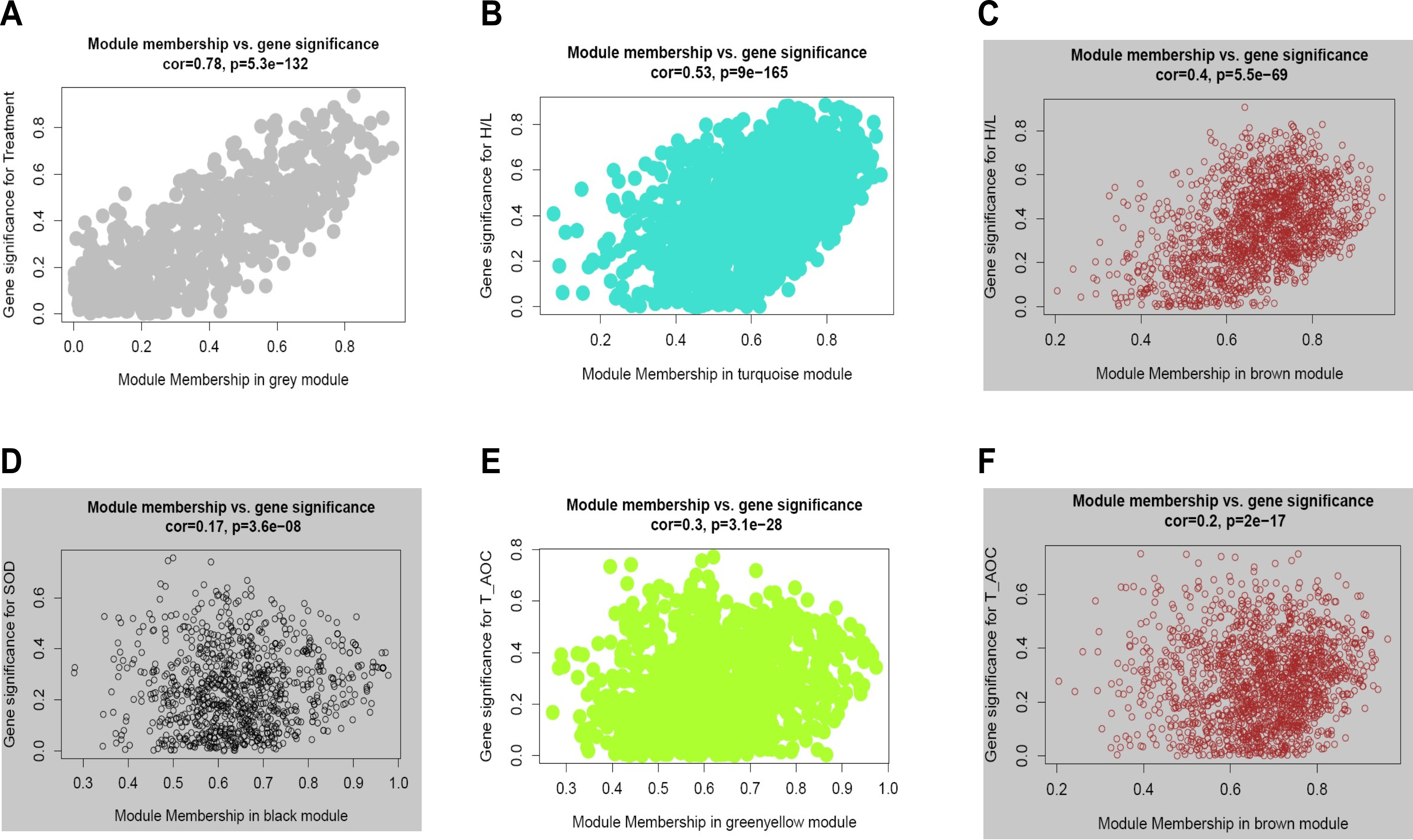

Supplement: Supplementary file 1 [file genes-13-00416-s001.zip › Supplement Figure S1.tiff]

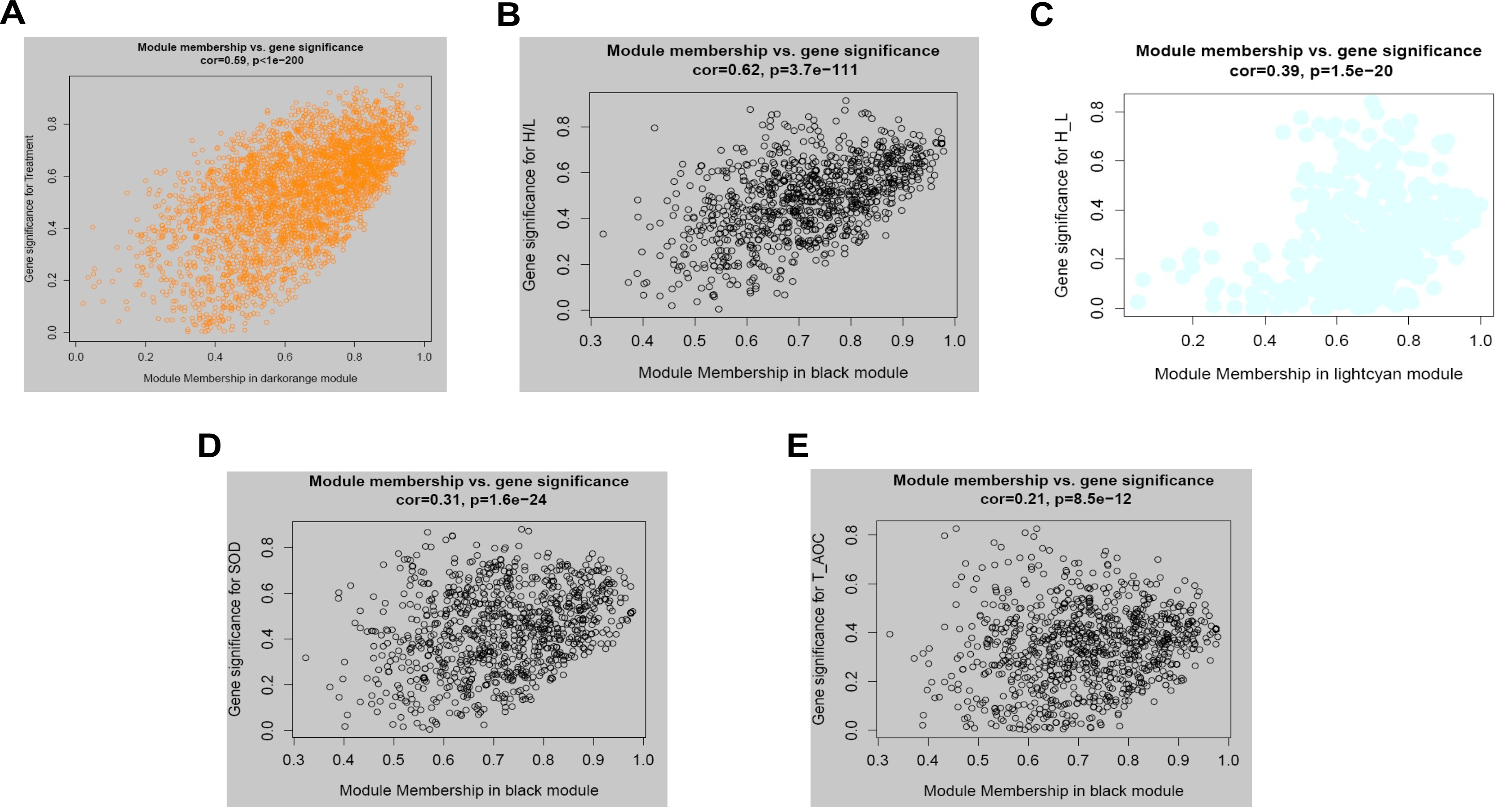

Supplement: Supplementary file 1 [file genes-13-00416-s001.zip › Supplement Figure S2.tiff]
